# MIP From *Legionella pneumophila* Influences the Phagocytosis and Chemotaxis of RAW264.7 Macrophages by Regulating the lncRNA GAS5/miR-21/SOCS6 Axis

**DOI:** 10.3389/fcimb.2022.810865

**Published:** 2022-04-29

**Authors:** Youfeng Shen, Jian Xu, Shenshen Zhi, Wenyan Wu, Yingying Chen, Qiang Zhang, Yan Zhou, Ze Deng, Wei Li

**Affiliations:** ^1^ Chongqing Precision Medical Industry Technology Research Institute, Chongqing, China; ^2^ Department of Clinical Laboratory, Chongqing Emergency Medical Center, Chongqing University Center Hospital, School of Medicine, Chongqing University, Chongqing, China; ^3^ Department of Pulmonary and Critical Care Medicine, Shengjing Hospital of China Medical University, Shenyang, China

**Keywords:** *Legionella pneumophila*, phagocytosis, chemotaxis, MIP, lncRNAGAS5

## Abstract

**Background:**

The intracellular pathogen *Legionella pneumophila* (*L. pneumophila*) is a causative agent of pneumonia and does great harm to human health. These bacteria are phagocytosed by alveolar macrophages and survive to replicate within the macrophages. Despite macrophage infectivity potentiator (MIP) protein serving as an essential virulence factor during the invasion process of *L. pneumophila*, the regulatory mechanism of MIP protein in the process of bacterial infection to host cells is not yet completely understood. This research thus aims to explore the interaction between MIP and macrophage phagocytosis.

**Methods:**

Through the experiment of the co-culture of RAW264.7 macrophages with different concentrations of MIP, the chemotactic activity of macrophages was detected and the phagocytosis was determined by a neutral red uptake assay. The expression of long noncoding RNA (lncRNA) GAS5, microRNA-21 (miR-21), and suppressor of cytokine signaling (SOCS)6 was determined by qRT-PCR. Target genes were detected by dual luciferase assay.

**Results:**

MIP could reduce the phagocytosis and improve the chemotaxis of RAW264.7 macrophages. The expression of both lncRNA GAS5 and SOCS6 was increased whereas the expression of miR-21 was decreased when macrophages were treated with MIP. Dual luciferase assay revealed that lncRNA GAS5 could interact with miR-21, and SOCS6 served as the target of miR-21. After GAS5 overexpression, the phagocytosis of RAW264.7 treated with MIP was increased whereas the chemotaxis was decreased. In contrast, the opposite results were found in RAW264.7 following GAS5 interference.

**Conclusions:**

The present results revealed that MIP could influence RAW264.7 macrophages on phagocytic and chemotactic activities through the axis of lncRNA GAS5/miR-21/SOCS6.

## Background

*Legionella pneumophila* (*L. pneumophila*) is a facultative intracellular and extracellular pathogen, with extensive distribution in various scenes of human daily life. It is typically transmitted by inhaling infected aerosols including contaminated water sprays or mists. As a result, it may opportunistically infect the alveolar macrophages leading to severe pneumonia called Legionnaires’ disease (Ludwig et al., 1994; Fonseca and Swanson., 2014) which was firstly described in the 1970s (Newton et al., 2010; Allegra et al., 2016) due to the outbreak among people attending a convention of the American Legion on a large scale. Given its different prevalence and routes of transmission, scientists around the world are making great efforts in investigating the pathogenesis and developing vaccines to effectively prevent and treat the most severe and common type of pneumonia (Fields et al., 2002). Pathogens have developed efficient ways to take advantage of the essential proteins of host molecules, along with molecular pathways to ensure their survival, differentiation, and replication in host cells (Leirião et al., 2004), and *L. pneumophila* is no exception (Dowling et al., 1992). *L. pneumophila* encodes specific virulence factors which have been evolved in the whole process of infection, including the adhesion of bacteria to host cells, the growth and proliferation of bacteria in host cells, and the stages of intercellular diffusion after proliferation (Cianciotto and Fields, 1992; Swanson and Hammer, 2000; Mengue et al., 2016). Heat shock protein 60 (Coers et al., 1999), major outer membrane protein (Zhu et al., 2010), and type IV pilus (Berger et al., 1994) have been reported as the virulence factors associated with adhesion and invasion of *L. pneumophila*. Furthermore, factors namely type IV B system effect factor (Berger et al., 1994; Pan et al., 2008), dot/icm complex loci (Berger et al., 1994; Pan et al., 2008), lvgA pathogenicity island (Ninio et al., 2009), and macrophage infectivity potentiator (MIP) (Cianciotto and Fields, 1992) contribute to intracellular survival and replication.

Being a population of immune cells that is plastic and pluripotent in the body, macrophages have evolved many defense strategies to combat infection with intracellular bacteria so that they can eliminate pathogens and maintain homeostasis of the internal environment (Weiss and Schaible, 2015). *L. pneumophilia* usually can get into the cytoplasm of macrophages by either winding phagocytosis or conventional phagocytosis, which is a process of endocytosis involving the macrophage cytoskeleton and actin (Quero et al., 2017). MIP is one of the recognized virulence factors contributing to the survival of *L. pneumophilia* in host cells. It can inhibit phagosome acidification and phagosome-lysosome fusion during phagocytosis, damage the killing function of macrophages to bacteria, and promote the proliferation of bacteria in phagosomes (Tijet et al., 2010; Juli et al., 2011). Whereas, its specific role and regulatory mechanism in this process are still poorly illustrated. The present study focused on the effect of MIP on macrophages and attempted to clarify the underlying mechanisms during the process. To achieve the specific objective of this study, the effect of MIP on macrophages was investigated to clarify the underlying mechanisms during the processes of phagocytosis and chemotaxis of RAW264.7 macrophage. The major finding of the research indicated a reduction of phagocytosis and an increase of chemotaxis for RAW264.7 macrophages after MIP treatment. Increasing studies have also reported that microRNAs might be involved in the process of macrophages responding to pathogen infection as critical regulators (Zhou et al., 2018). Long noncoding RNAs (lncRNAs) are considered to be able to modulate gene expression in the polarization of macrophages (Mosser and Edwards, 2008), which is an important step for macrophages to identify invasive pathogens and activate downstream immune responses. Moreover, the suppressor of cytokine signaling 6 (SOCS)6 is a protein encoded by the SOCS6 gene which encodes a member of the STAT-induced STAT inhibitor, also known as a suppressor of cytokine signaling (Alexander and Hilton, 2004). In this study, we aimed to investigate the effect of MIP on phagocytosis, the chemotactic activity of RAW264.7, and the regulation of lncRNA GAS5/microRNA-21 (miR-21)/SOCS6 axis.

## Methods

### Cell Growth Condition

RAW264.7 cells were selected as the appropriate macrophage model as such cells were capable of performing pinocytosis and phagocytosis. RAW264.7 cell lines were bought from Biomedicine Biotech (Chongqing, China) and preserved in Dulbecco’s modified Eagle’s medium (Shanghai Basalmedia Technologies Co. Ltd.) together with the following components to the base medium including fetal bovine serum (HyClone, Logan, UT, USA) at a final concentration of 10%, 2 mM l-glutamine (Thermo Fisher Scientific) and 100 U/ml penicillin (Solarbio, Beijing, China). Cell incubation was performed at 37°C with 5% CO_2_.

### Cell Counting Kit-8 Assay

The toxicity of MIP to RAW 264.7 macrophages and 50% inhibitory concentration (IC50) were detected by the Cell Counting Kit-8 (CCK-8) assay. Cells in the logarithmic phase were neutralized by conventional digestion, resuspended evenly using a fresh culture medium, and the relative cell activity was calculated. The cell concentrations were adjusted to 6 × 10^4^ cells/ml. The cell suspension was added to 96-well plates, 100 µl for each well (approximate 6 × 10^3^ cells/well), and then incubated overnight at 37°C with 5% CO_2_. The MIP solution at different concentrations was subsequently transferred to the plates, 100 µl per well. Meanwhile, blank (the culture medium without cells adding CCK-8) and control (the culture medium seeding cells without CCK-8) wells were set with three multiple wells for each group. Following MIP treatment at varying concentrations for 24 h, 20 µl of CCK-8 reagent (Engreen Biosystem, Beijing, China) was added to each well and incubated for 3 h at 37°C. The optical density (OD) was measured by a microplate reader (Beckman Coulter, Fullerton, CA, USA) at 450 nm. Each concentration in the 96-well plates was detected in triplicates, and the experiments were repeated independently at least three times. The IC50 was calculated using GraphPad Prism software (Version 8.0.2). According to the obtained IC50 value, three concentrations of MIP were applied in the subsequent experiments: 0.1 × IC50 for the low-dose group, 0.2 × IC50 for the medium-dose group, and 0.4 × IC50 for the high-dose group.

### Plasmid and Antibody

The present study constructed the recombinant plasmid pET-MIP of *L. pneumophila* which was kept at −20°C for later use. The plasmids of backbone vectors including pGL3-basic, pRL-TK, pcDNA3.1(+), and pRNAT-U6.1 were bought from Promega (Beijing, China).

### Transwell Assay

Transwell assay was used to detect the effect of MIP on chemotaxis of RAW264.7 macrophages under the instructions of a 24-well Transwell system from Corning, NY, USA. A volume of 100 µl medium containing 6 × 10^4^ cells was plated to each upper insert and another 500 µl medium was added to the lower insert. The wells containing MIP were set as the treated group, and the lower wells with MIP-free medium were taken as the control. There were six replicates for each well. The inserts were added into the wells without bubbles and incubated at 37°C with 5% CO_2_. After 24, 48, and 72 h, the upper insert was removed and washed using PBS twice. The cells that failed to pass through the membrane were removed gently. The inserts were transferred to wells containing 500 μl of paraformaldehyde and fixed, then subsequently turned upside down 30 min later and dried naturally. Next, the samples were placed into a well containing 0.1% crystal violet (Solarbio, Beijing, China), stained avoiding light for 15 min, rinsed with crystal violet away from the inserts with ddH_2_O, dried, and photographed under a microscope. To figure out the chemotaxis index, bound crystal violet was eluted using 33% acetic acid and the OD value of the eluent was measured at 570 nm. The chemotaxis index was calculated as follows: Chemotaxis index (%) = (OD of treatment/OD of control) × 100%.

Transwell assay was also employed to determine the effect of MIP on chemotaxis of RAW264.7 macrophages transfected by overexpressed GAS5 and interfered with GAS5, respectively. Plasmids included pcDNA3.1(+)-GAS5 and pRNAT-U6.1-siGAS5 according to the modification methods suggested by the manufacturer. RAW264.7 cells were subsequently transfected using TransFast™ Transfection Regent (Promega E2431). Following 24 h transfection, the cells were collected for Transwell assay according to the manufacturer’s instructions.

### Neutral Red Uptake Assay

The effects of MIP on the phagocytosis of RAW264.7 cells were evaluated by the neutral red uptake assay based on the protocol previously published by Guillermo Repetto et al. in 2008 (Aderem and Underhill, 1999). Following the addition of 100 μl fresh medium containing about 6 × 10^4^ RAW264.7 cells into 96-well plates, the cells were incubated overnight at 37°C with 5% CO_2_. The cell suspension was supplemented with 100 μl of medium containing different concentrations of MIP subsequently. Meanwhile, the cell wells containing merely 100 μl medium were set as the control group. Following 24 h incubation at 37°C with 5% CO_2_, the medium was removed and followed by two cycles of PBS washing. Following preheating the neutral red staining medium at 37°C (Solarbio, Beijing, China), 100 μl of the solution was added to each well after incubated at 37°C for 2 h, then the neutral red medium was removed and the cells were washed using 150 μl PBS by gentle aspiration 3 times. An addition of 150 μl neutral red destaining solution containing 50% ethanol, 1% glacial acetic acid, and 49% deionized water was supplied to each well. The plates were then vibrated quickly on a shaker for 10 min so that the cells could be completely suspended. Consequently, the OD value was detected using a microplate reader at 540 nm, with a cell-free blank as a reference. The morphological changes of cells were visualized and reported using a phase-contrast inverted microscope. The experiment had at least three repeats to ensure the data were statistically significant.

The measurement results were in agreement with chemotaxis. The effect of MIP on the phagocytic activity of RAW264.7 macrophages transfected by overexpressed GAS5 and interfered with GAS5 were detected simultaneously. The assays of transfection and neutral red uptake were conducted as previously described.

### Total RNA Isolation and qPCR

Following MIP treatment for 12, 24, and 36 h, RAW264.7 cells were collected for gene expression analysis that was hypothesized to link to phagocytosis and chemotaxis. Total RNA was extracted using TRIZOL reagent (Takara, Dalian, China) following the instructions of the manufacturer; 2 μg RNA from each sample was collected for later use. To compare the expression levels of miR-21 in treated and untreated cells, cDNA synthesis was carried out according to the protocol of One Step miRNA cDNA Synthesis Kit (HaiGene, Harbin, China), in which the reverse transcription was performed with a modified oligo (dT) primer after the polyadenylation of miRNAs (Balcells et al., 2011). The primers of miR-21 were listed as follows: miR-21-5p-F: TCAGTAGCTTATCAGACTGATG; and miR-21-5p-R: TTACCTAGCGTATCGTTGAC. U6 was selected as an internal control, and its primers were U6-F: GTGCTCGCTTCGGCAGCA and U6-R: GAACGCTTCACGAATTTGCG. To analyze GAS5 and SOCS6 expression, cDNA was synthesized using MMLV Reverse Transcriptase (Takara, Dalian, China), and gene expression levels were normalized using the gene GAPDH. The primers included the following: GAS5-F: GGCAAAGGAGGATGAAGGCT; GAS5-R: AGCAAGCCAGCCAAATGAAC; SOCS6-F: CTATTGCAGAGTTGTAAGCTTG, SOCS6-R: CTGCCATCTAATATAGATGCAC; GAPDH-F: TGTGTCCGTCGTGGATCTGA, and GAPDH-R: TGCTGTTGAAGTCGCAGGAG. qPCR was subsequently performed on the 7300 Real-Time PCR System from Applied Biosystems (Foster City, CA, USA) using the SYBR green method as instructed (95°C for 10 s, 1 cycle; 95°C for 5 s and 60°C for 30 s, 40 cycles in total). All reactions were carried out with three replicates, and the average Ct value of each group was used to calculate the relative expression level. The delta–delta Ct method was applied, and the calculation formulas were ^△^Ct = Ct (target gene) − Ct (action gene) and ^△△^Ct = ^△^Ct (treatment) − ^△^Ct (control).

### Western Blot

The protein SOCS6 was firstly extracted from RAW264.7 and then detected using Western blot. After the addition of protein lysis buffer, the protein was placed on ice for 30 min and centrifuged for supernatant collection. The protein was denatured at 100°C for 8 min, isolated with 13% SDS-polyacrylamide gel electrophoresis, followed by membrane transference for 7.5 min, and blocked with skim milk for 1 h. The SOCS6 antibody (PA5-50263, Thermo Fisher Scientific, Shanghai, China) was added and incubated overnight at 4°C. After membrane washing with TBST, the GAPDH antibody (1:10,000, AC002, ABclonal, Wuhan, China) was added and incubated at 37°C for 1 h. The result was scanned, and the OD value of the band was analyzed for quantification. The OD ratio of the target band was employed to GAPDH as the relative expression of the target protein.

### Dual-Luciferase Reporter Assay

According to the results of expression and bioinformatics prediction, SOCS6 was the putative target of miR-21 (TargetScan http://www.targetscan.org). The 3’ untranslated regions (UTR) of SOCS6 were selected to verify its interactions with miR-21 using a dual-luciferase reporter assay. Similar procedures were repeated to verify the relationship between lncRNA and miR-21. pGL3-basic containing firefly luciferase activities were selected as the backbone to construct work vector, and pRL-TK-Rluc containing Renilla luciferase activities were chosen as an internal control to eliminate the influence of experimental errors. The following five groups of plasmids were prepared for later use including pGL3-basic-Luc-3’UTR-SOCS6, mimics-miR-21, pGL3-basic-Luc-3’UTR-SOCS6+mimics-miR-21, pGL3-basic-Luc-GAS5, and pGL3-basic-Luc-GAS5 + mimics-miR-21. The five prepared RAW264.7 cell groups were cotransfected with pRL-TK-Rluc using TransFast™ Transfection Reagent (Promega E2431) at a 3:1 ratio, respectively. Cells were collected 24 h after the cotransfection, and the firefly and Renilla luciferase activities were determined by a dual luciferase reporting assay from Promega as per the manufacturer’s instructions. The experiment was repeated three times for data collection, and the average value was calculated for further analysis. The luciferase activity of firefly was normalized *via* internal control and then analyzed by GraphPad Prism software (Version 8.0.2).

### Statistical Analysis

All data were analyzed with SPSS 23.0 software (SPSS, USA) and indicated as mean values ± SD. Methods of one-way ANOVA or Student’s *t*-test were used to examine statistical significance through a comparison of mean values in different groups. *p* < 0.05 was considered statistically significant which was labeled by an alphabetic-based notation (a, b, c, and d), to mark the significant differences in figure legends.

## Results

### MIP Increases the Chemotaxis and Inhibits the Phagocytosis of RAW264.7

**Figure 1:**
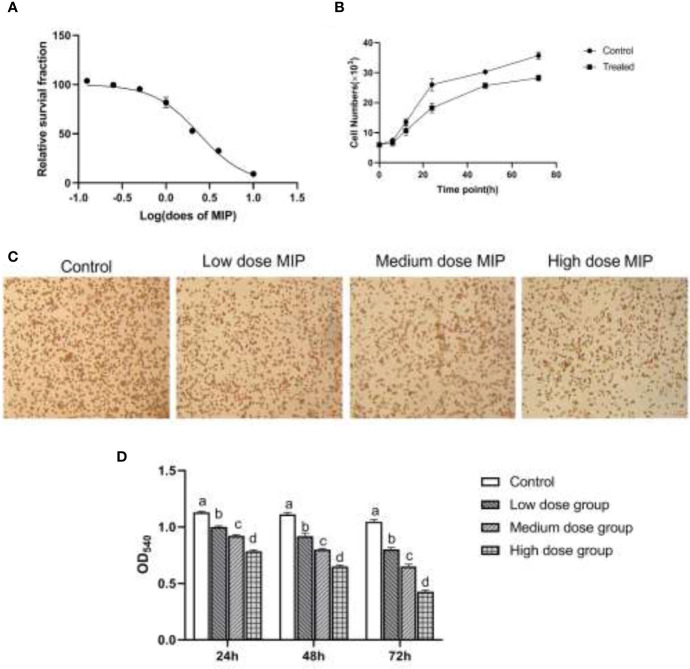
MIP inhibits the proliferation and phagocytic activity of RAW264.7. **(A)** A dose-response study of MIP indicated IC50 as 2.384 ng/mL. **(B)** The proliferous capacity of RAW264.7 upon the treatment of MIP. **(C)** The cytological morphology of RAW264.7 from neutral red uptake assay at 24 h in the four groups (control cells; cells treated with 0.24 ng/ml MIP; cells treated with 0.48 ng/ml MIP; cells treated with 0.96 ng/ml MIP); **(D)** Semi-quantitative analysis of the phagocytosis of RAW264.7. The significant differences were marked by the alphabetic notation (a–d) by using ANOVA. In the control and different concentration treatment groups, the control is marked with the letter a; in the low-dose group, an average that is significantly different from it is marked with the letter b, otherwise, letter a and continuously marked with letters c and d.

To investigate whether MIP had cytotoxic effects on the RAW264.7 macrophages, the CCK-8 assay was used to determine cell viability. The cell survival fraction demonstrated that cell viability was affected after MIP treatment with a half maximal inhibitory concentration (IC50) at 2.384 ng/ml. Significant changes in cytotoxicity were observed in the cells treated with MIP instead of the untreated and varied with the increase of MIP concentrations (**Figure 1A**) and time axis (**Figure 1B**). In line with the IC50, different concentrations of MIP were set as three corresponding work doses: low (0.1 × IC50 = 0.24 ng/ml), medium (0.2 × IC50 = 0.48 ng/ml), and high (0.4 × IC50 = 0.96 ng/ml) doses.

**Figure 2:**
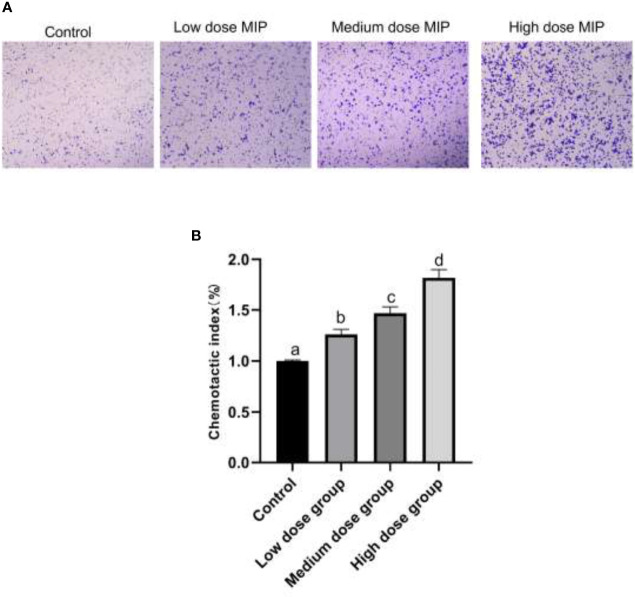
MIP enhances the chemotaxis of RAW264.7. **(A)** The cytological morphology of RAW264.7 cells that migrated across the membrane in the MIP-free medium, low-dose MIP, medium-dose MIP, and high-dose MIP. **(B)** Chemotactic index of RAW264.7. The significant differences were marked by the alphabetic notation (a–d) by using ANOVA. In the control and different concentration treatment groups, the control is marked with the letter a; in the low-dose group, an average that is significantly different from it is marked with the letter b, otherwise, letter a and continuously marked with letters c and d.

RAW264.7 was cocultured with different doses of MIP for 24 h; the chemotaxis and phagocytosis of RAW264.7 macrophages were detected by Transwell assay and neutral red uptake assay, respectively. The results of cytological observation and statistical data analysis revealed that MIP could reduce the phagocytic activity of RAW264.7 macrophages (**Figures 1C, D**) and improve chemotaxis (**Figures 2A, B**).

Taken together, the RAW 264.7 cells cocultured with MIP showed a lower ability for neutral red uptake than cells from the control group (*p* < 0.05), and the changes were in a time- and concentration-dependent manner. By comparison, chemotaxis of RAW264.7 cells indicated an increase in a time- and concentration-dependent manner after the treatment of MIP.

### lncRNA GAS5 and miR-21 Can Be Downregulated While SOCS6 Can Be Upregulated in RAW264.7 Cells Treated by MIP

The phagocytosis of pathogens in RAW264.7 macrophage is mediated by a series of gene transcriptional regulations in which noncoding RNAs are to be crucial posttranscriptional regulators of macrophage discrimination in bacterial infection (Wang et al., 2020).

**Figure 3:**
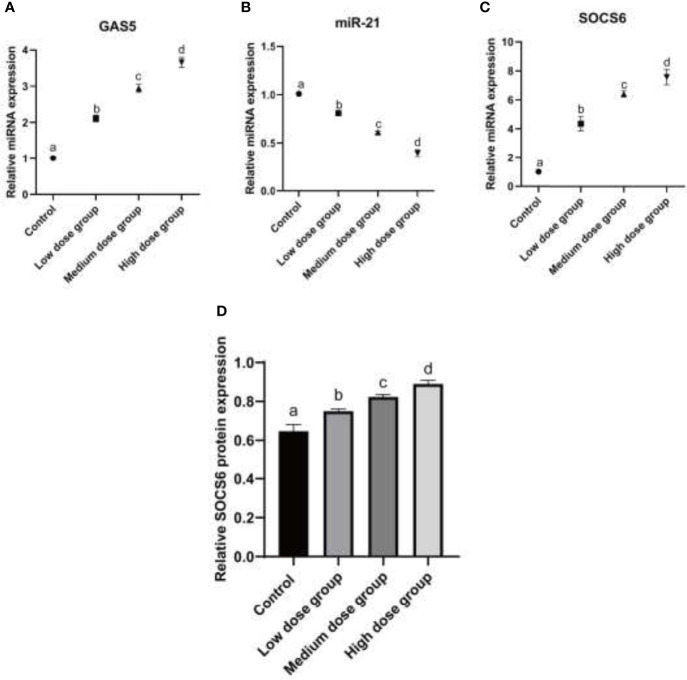
Analysis of expression of GAS5/miR-21/SOCS6 in RAW264.7 treated with MIP. **(A)** Relative mRNA expression analysis of GAS5 at 24 h treated by different concentrations of MIP. **(B)** Relative mRNA expression analysis of miR-21 at 24 h treated by different concentrations of MIP. **(C)** Relative mRNA expression analysis of SOCS6 at 24 h treated by different concentrations of MIP. **(D)** Relative protein expression of SOCS6 was detected by WB. The significant differences were marked by the alphabetic notation (a–d) by using ANOVA. In the control and different concentration treatment groups, the control is marked with the letter a; in the low-dose group, an average that is significantly different from it is marked with the letter b, otherwise, letter a and continuously marked with letters c and d.

To further understand how MIP affects the working mechanism during the process of *L. pneumophila* infection, the expression pattern of lncRNA GAS5, miR-21, and its putative target SOCS6 mRNA and protein were detected. The results displayed that the expression of both lncRNA GAS5 and SOCS6 was increased while the expression of miR-21 was decreased (**Figures 3A–D**).

### miR-21 Binds to lncRNA GAS5 While It Specifically Targets SOCS6

**Figure 4:**
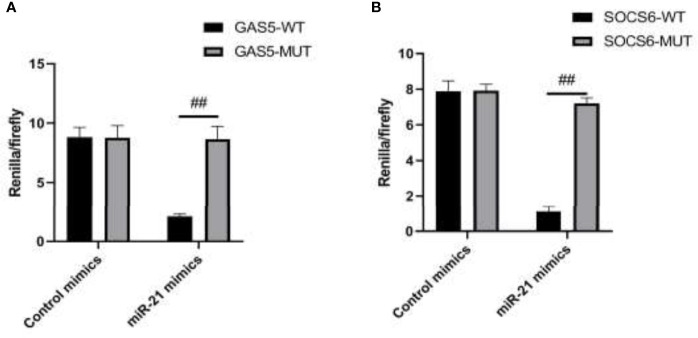
The targeting relationship of GAS5/miR-21/SOCS6 is detected by a dual luciferase reporting system. **(A)** The targeting relationship of GAS5/miR-21 was detected by a double luciferase reporting system. **(B)** The targeting relationship of miR-21/SOCS6 was detected by a dual luciferase reporting system. Statistical significance was determined by the Student’s *t*-test (^##^ *p* < 0.01).

SOCS6 is a protein encoded by the gene SOCS6. The gene encodes a member of the STAT-induced STAT inhibitor (SSI) and is also known as a SOCS. Using the target gene prediction software from the public databases TargetScan, PicTar, and MiRBase, we found that there were eight consecutive complementary bases in the 3’UTR region of SOCS6 and miR-21, which might be the targets. The working mechanism after the treatment of MIP was explored by dual-luciferase reporter assay. The results of the assay demonstrated that miR-21 could specifically target 3’UTR of SOCS6 and lncRNA GAS5 could bind to miR-21 at the structure to affect its regulation (**Figures 4A, B**). 3’UTR could regulate several biological processes including mRNA stability, subcellular localization, and translation. Numerous studies have shown that 3’UTR is closely related to human diseases and livestock production performance.

### MIP Influences the Phagocytosis and Chemotaxis of RAW264.7 Macrophages by Regulating the lncRNA GAS5/miR-21/SOCS6 Axis

**Figure 5:**
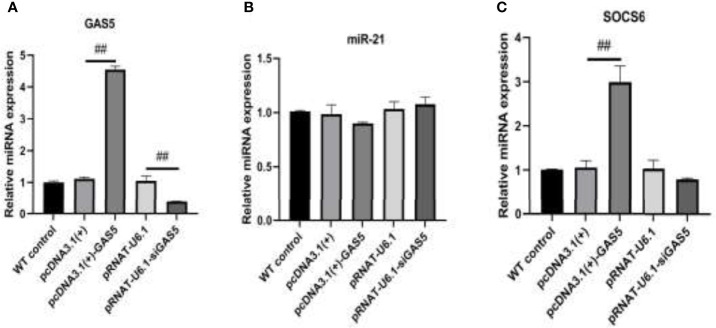
The expression of GAS5/miR-21/SOCS6 is detected after overexpression and interference with GAS5. **(A)** The expression level of GAS5. **(B)** The expression level of miR-21. **(C)** The expression level of SOCS6. Statistical significance was determined by the Student’s *t*-test (^##^ *p* < 0.01).

**Figure 6:**
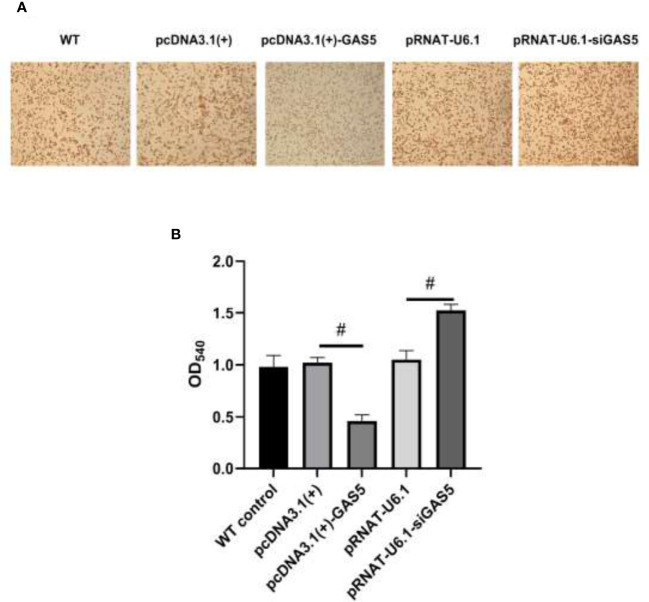
GAS5 inhibits the phagocytic activity of RAW264.7. **(A)** The cytological morphology of RAW264.7 from neutral red uptake assay at 24 h in the five groups. **(B)** Semiquantitative analysis of the phagocytosis of RAW264.7. Statistical significance was determined by the Student’s *t*-test (^#^ *p* < 0.05).

**Figure 7:**
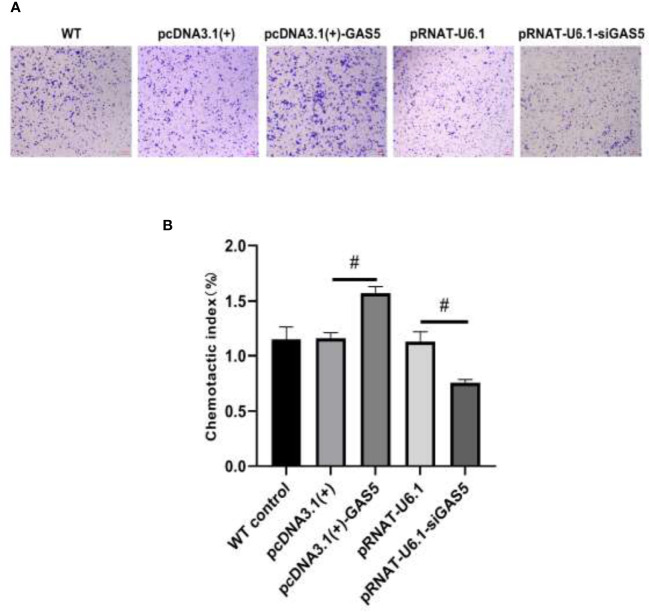
GAS5 enhances the chemotaxis of RAW264.7. **(A)** The cytological morphology of RAW264.7 cells that migrated across the membrane. **(B)** Chemotactic index of RAW264.7. Statistical significance was determined by the Student’s *t*-test (^#^ *p* < 0.05).

The plasmids pcDNA3.1(+)-GAS5 and pRNAT-U6.1-siGAS5 were used to induce GAS5 overexpression and silence. After overexpression of GAS5 was induced in RAW264.7, the expression of GAS5 and SOCS6 was increased significantly, while the expression of miR-21 had no significant difference among each group; then after interference with GAS5, the expression of GAS5 and SOCS6 in RAW264.7 was decreased, and the expression of miR-21 was comparable in empty vector pRNAT-U6.1 and pRNAT-U6.1-siGAS5 (**Figures 5A**–**C**). After overexpressing and interfering with GAS5, the macrophages were treated with MIP to observe the effect on the chemotaxis and phagocytosis. After transfection with overexpressed GAS5, an increase of phagocytosis (**Figures 6A, B**) and a decrease of chemotaxis (**Figures 7A, B**) of RAW264.7 were revealed after MIP treatment whereas an opposite result was found after interfering with GAS5. The research results proved that MIP could affect the phagocytic and chemotactic activities of RAW264.7 macrophage through the axis of lncRNA GAS5/miR-21/SOCS6.

## Discussion

*L. pneumophila* is an intracellular parasitic bacteria that has evolved multiple effective strategies to escape from phagocytes. Consequently, it can survive in cells for a long time and infect the human body. The well-recognized Legionnaires’ disease, a severe form of pneumonia, is caused by the bacteria. Alveolar macrophages are the first immune cells that respond to inhaled pathogens when *L. pneumophila* evades the human body. The macrophages are capable of antigen presentation, phagocytosis, and chemotaxis, while *L. pneumophila* can be phagocytosed by macrophages instead of being eliminated. These bacteria survive, replicate, and reproduce in macrophages. They escape from the killing mechanism of macrophages, which is the main reason for its great pathogenicity. Although little is known about the pathogenesis of *L. pneumophila*, some virulence factors have been reported as the development targets for vaccines or therapeutic methods. Furthermore, MIP is a known virulence factor in *L. pneumophila* and is reported to be essential for invasion and proper intracellular establishment of *L. pneumophila* in macrophages, thereby contributing to the survival of *L. pneumophila (* Wintermeyer et al., 1995; Wieland et al., 2002; Shevchuk et al., 2011). Unfortunately, how MIP affects macrophages with what underlying mechanism remains unclear. The present study focused on the exploration of how MIP of *L. pneumophila* affected the phagocytosis and chemotaxis of RAW264.7 macrophages. MIP, the macrophage infection enhancer protein, was the specific protein component of legionella and one of the virulence factors related to legionella’s survival.

Macrophages belong to one of the phagocytes derived from monocytes; they participate in both nonspecific defense (innate immunity) and specific defense (cellular immunity) in vertebrates (Wintermeyer et al., 1995; Aderem and Underhill, 1999; Wieland et al., 2002; Balcells et al., 2011; Shevchuk et al., 2011; Wang et al., 2020). The effect of phagocytosis and chemotaxis of macrophages can be changed by altering the patterns of transcription so that the invaded intracellular parasitic bacteria can be eliminated (Alexander and Hilton, 2004; Mosser and Edwards, 2008; Tijet et al., 2010; Juli et al., 2011; Weiss and Schaible, 2015; Quero et al., 2017; Zhou et al., 2018).

This study, for the first time, investigated the expression of lncRNA GAS5 and relevant microRNA miR-21 in macrophages stimulated by MIP. We observed reduced phagocytosis and enhanced chemotaxis in RAW264.7 cells cultured with MIP. MIP serves as a virulence factor of legionella, it attenuates the recognition and clearance of the bacteria, leads to cell apoptosis and necrosis, releases bacteria, and improves its survival rate by inhibiting the phagocytosis of macrophages. Some studies have found that lncRNA GAS5 siRNA transfection promotes the uptake of *Escherichia coli* by macrophages and regulates the phagocytosis of macrophages (Ahmad et al., 2020). Hu et al. have demonstrated that overexpression of GAS5 *in vitro* promotes the polarization of macrophages (RAW) towards the M1 phenotype by inducing nitric oxide synthase (iNOS), interleukin-1β, and tumor necrosis factor-alpha, and reduced levels of GAS5 in diabetic wounds appear to enhance healing by promoting the transition of M1 macrophages to M2 macrophages (Hu et al., 2020). Moreover, the study has shown that exogenous lncRNA GAS5 regulates macrophage apoptosis in atherosclerosis (Chen et al., 2017). MiR-21 serves as a GAS5 target; the negative regulation of miR-21 by GAS5 has been reported in previous studies (Yu et al., 2020; Cong et al., 2020; Jiang and Du, 2022). Our results indicated that following the treatment of virulence factor MIP, lncRNA GAS5 was upregulated following MIP treatment in a dose-dependent manner whereas miR-21 was downregulated. The putative target gene SOCS6 of miR-21 obtained from the TargetScan (a miRNA target gene prediction database) which has also been previously shown to regulate SOCS6 is upregulated in RAW264.7 after MIP treatment (Li et al., 2015; Liu et al., 2018). SOCS6 has been identified as a member of the SOCS protein family. Its correlation with *L. pneumophila* infection has not been reported before. However, the role of SOCS in the macrophages in viral and bacterial infections has been reported in recent studies (Labuzek et al., 2012; Duncan et al., 2017). The bacterial pathogens can evade host immune defenses by employing SOCS protein to manipulate cytokine receptor signaling transduction (Baetz et al., 2007). Thus, overexpression of SOCS is implicated in immune escape and disease progression. Especially, SOCS3 has been induced to regulate macrophage activation by mediating tyrosine phosphorylation of STAT1 in the persistent infections of *L. monocytogenes* (Okada et al., 2009). Furthermore, overexpression and interference vector of GAS5 was transfected in RAW264.7; the phagocytosis was decreased markedly while chemotaxis was enhanced significantly. The influence of MIP by mediating the lncRNA GAS5/miR-21/SOCS6 was further demonstrated.

## Conclusions

The data of the present study suggested that MIP might impair phagocytosis and enhance chemotaxis through the axis of lncRNA GAS5/miR-21/SOCS6 so that it could modulate the function of macrophages infected by *L. pneumophila*. Our investigation results contribute to providing a new target for developing effective prevention and treatment methods against *L. pneumophila* infection.

## Data Availability Statement

The datasets used and/or analysed during the current study are available from the corresponding author on reasonable request.

## Author Contributions

YS and JX designed the study. YZ and WL designed this study and drafted the manuscript. SZ, WW and YC analyzed and interpreted the experimental data. YS, JX, SZ, WW, YC and QZ performed the Transwell, CCK-8, neutral red uptake, and double luciferase assays. YZ performed qRT-PCR and western blot. YS, JX and WL contributed to the revision of the manuscript. All authors read and approved the final manuscript.

## Conflict of Interest

The authors declare that the research was conducted in the absence of any commercial or financial relationships that could be construed as a potential conflict of interest.

## Publisher’s Note

All claims expressed in this article are solely those of the authors and do not necessarily represent those of their affiliated organizations, or those of the publisher, the editors and the reviewers. Any product that may be evaluated in this article, or claim that may be made by its manufacturer, is not guaranteed or endorsed by the publisher.
